# Evaluation of compound NXY-059 on cognitive impairment caused by sepsis

**DOI:** 10.1186/cc13006

**Published:** 2013-11-05

**Authors:** Nathália S Oliveira, Monica F Pereira, Mariana GAT Cunha, Silvio CA Júnior, Rachel N Gomes, Patrícia A Reis, Robert Floyd, Hugo CCF Neto

**Affiliations:** 1 Instituto Oswaldo Cruz, Fiocruz, Rio de Janeiro, RJ, Brazil; 2 Oklahoma Medical Research Foundation, Oklahoma City, OK, USA

## Background

Nitrones are a class of molecules whose main effect on biological systems is their antioxidant action. Some studies showed a neuroprotective effect in ischemia models and neurodegenerative diseases. Those diseases presented an inflammatory profile that leads the production of reactive oxygen species. This characteristic can generate brain injuries, which can affect areas related to memory consolidation. Sepsis is a pathology that forms an inflammatory response, which causes encephalopathy creating cognitive impairment. Therefore, the present study has the aim to evaluate the effect of the compound NXY-059 on the cognitive impairment caused by encephalopathy like sepsis.

## Materials and methods

For the assays, mice Swiss Webster male (22 to 28 g, *n *= 15 per group) were submitted to the CLP method and treated with antibiotics (10 mg/kg, i.p.) for three consecutive days (6, 24 and 48 hours) and with NXY-059 (50 mg/kg, i.p.) for five consecutive days (6, 24, 48, 72 and 96 hours after the surgery). At 24 and 48 hours, a gravity score was made to determine the level of sepsis and the percent of survival was assessed until 144 hours. After 4 hours fast, the glucose levels were also measured 24 and 48 hours after CLP performance. The cognitive impairment was evaluated through the open field method on the 15th (training) and 16th (test) day after the surgery.

## Results

Our results show that treatment with NXY-059 did not offer a protective effect on mortality and the animals developed moderated sepsis according to the gravity score at 24 hours (4 to 6). At 48 hours, the animals recovered for slight sepsis (2 to 3). The glucose levels were slightly restored at 48 hours for the animals treated with the compound. In the cognitive impairment analysis, we observed a as reduction (*P *< 0.05) in the numbers of crossing and rearings for the animals treated with NXY-059 when compared with animals treated with vehicle (saline).

## Conclusions

According to these results, we can suggest that treatment with NXY-059 offered protection against cognitive impairment generated by sepsis.

